# Connectivity dynamics in Irish mudflats between microorganisms including* Vibrio* spp., common cockles *Cerastoderma edule*, and shorebirds

**DOI:** 10.1038/s41598-021-01610-x

**Published:** 2021-11-12

**Authors:** Sara Albuixech-Martí, Sharon A. Lynch, Sarah C. Culloty

**Affiliations:** 1 School of Biological, Earth and Environmental Sciences, University College Cork, Cork, VGV5+95 Ireland; 2 Aquaculture and Fisheries Development Centre, University College Cork, Cork, VGV5+95 Ireland; 3 MaREI Centre for Climate, Energy and Marine, Environmental Research Institute, University College Cork, Cork, VGV5+95 Ireland

**Keywords:** Marine biology, Community ecology, Bacterial infection

## Abstract

Shellfish, including the key species the common cockle *Cerastoderma edule,* living and feeding in waters contaminated by infectious agents can accumulate them within their tissues. It is unknown if microbial pathogens and microparasites can subsequently be transmitted via concomitant predation to their consumers, including shorebirds. The objective of this study was to assess if pathogens associated with *C. edule* could be detected seasonally in the faeces of shorebirds that feed on *C. edule* and in the physical environment (sediment) in which *C. edule* reside, along the Irish and Celtic Seas. Two potentially pathogenic global groups, *Vibrio* and Haplosporidia, were detected in *C. edule*. Although Haplosporidia were not detected in the bird faeces nor in the sediment, identical strains of *Vibrio splendidus* were detected in *C. edule* and bird faecal samples at sites where the oystercatcher *Haematopus ostralegus* and other waders were observed to be feeding on cockles. *Vibrio* spp. prevalence was seasonal and increased in *C. edule* and bird faecal samples during the warmer months, possibly due to higher seawater temperatures that promote the replication of this bacteria. The sediment samples showed an overall higher prevalence of *Vibrio* spp. than the bird faecal and *C. edule* samples, and its detection remained consistently high through the sites and throughout the seasons, which further supports the role of the sediment as a *Vibrio* reservoir. Our findings shed light on the fact that not all pathogen groups are transmitted from prey to predator via feeding but bacteria such as *V. splendidus* can be. As most of the wading birds observed in this study are migratory, the results also indicate the potential for this bacterium to be dispersed over greater geographic distances, which will have consequences for areas where it may be introduced.

## Introduction

Mudflats are coastal wetlands that form in sheltered intertidal areas such as bays and estuaries, where sediments have been deposited by tides or rivers. Mudflats support a large population of wildlife and are a key habitat for many migratory shorebirds, as well as for certain species of molluscs, crabs, and fish^1^. The common cockle *Cerastoderma edule* is widely distributed in the Atlantic and one of the main non-cultured bivalve species harvested in western European bays and estuaries, where population densities of 10,000 per m^2^ have been recorded^2^, making them a useful model species for this study. Furthermore, common cockles play a key role as an ecosystem engineer, controlling or influencing processes such as bioturbation and water filtration, increasing the productivity of sedimentary habitats, which underpin marine food webs and biodiversity^3–5^. In the marine food chain, *C. edule* is an important food source and are a link between primary producers (phytoplankton, phytobenthos) and consumers such as crabs, shrimps, fish and birds^4^. Cockles, inhabiting the intertidal, are easily available to a variety of predators since they are found only a few millimetres beneath the sediment surface, they may even emerge at the surface when abundance is high, because of sediment erosion, or when they are stressed, such as by oxygen deficiency or trematode infection^6^. One of the main consumers feeding on up to 300 cockles by day is marine bird populations^3^, whose feeding ecology may shape their diverse and multiple endoparasitic fauna^7^, responsible for significant effects on marine birds^8^. Trophically transmitted parasites and pathogens are central elements in most aquatic food webs, given that foraging behaviour and diet facilitate the transmission of a variety of pathogens and endoparasites through marine invertebrate and vertebrate species^9,10^. Concomitant predation is the most common way for parasites and pathogens to become prey, which occurs when an infected host is eaten by a predator. Because of the high productivity of aetiological agents, their unique nutritional composition and their pathogenicity in hosts, their consumption affects food web structure, energy transfer and disease dispersals^11^.

Although parasitism contributes significantly to the biodiversity of mudflat ecosystems, the composition of parasites in the food web of the mudflat ecosystems has been understudied^1^. Cockles inhabiting those habitats can accumulate agents that are potentially pathogenic^12^ and act as a reservoir for subsequent infection of other species^5^. Cockles are hosts to a diverse range of macroparasites^13^, but may be also infected by a range of pathogens including protists, bacteria and viruses^14^. In turn, Newman et al.^15^ confirmed that infectious diseases are an important cause of mortality events and individual bird deaths, especially among coastal and freshwater aquatic birds, pointing out bacterial infections as a frequent cause of mass mortality events.

A wide range of bacterial infections occurs in free-ranging birds^8^, with many copiotrophic bacteria, such as *Vibrio* spp., widely distributed in estuarine and coastal ecosystems^16,17^. A large number of *Vibrio* species are associated with marine organisms like fish, molluscs and crustaceans, in commensal or pathogenic relations^18^. Some species such as *Vibrio aestuarianus*, *Vibrio splendidus* or *Vibrio tapetis* have been associated with mortalities of different molluscan species, seriously affecting their culture and causing high losses in hatcheries as well as in natural beds^19–24^. For other species, ecological importance has been demonstrated, such as *Vibrio crassostreae, Vibrio breoganii, Vibrio celticus*, which form part of the molluscan microbiota^18^, or *V. mediterranei*, found in the microbiota of marine fish^25^. In addition, *Vibrio* bacteria can persist in high abundance and/or can be found during cold and unfavourable environmental conditions in the sediment compartment^26,27^. Azandégbé et al.^28^ reported the occurrence of *V. aestuarianus* in sediment at two *C. gigas* farms in France and suggested that this bacterium might subsist during the cold seasons in the sediment and rise again under favourable conditions.

The haplosporidian protists are also widespread and have been associated with some of the most serious epizootic mortalities of commercially important bivalves, including cockle population crashes in The Netherlands and UK^14,29–32^. A number of studies have reported the detection of *Minchinia*^14,30,33–35^ and *Haplosporidium* in cockles^30,36^, and *Urosporidium* parasitizing trematodes or turbellaria infecting cockles^37^. Cockles are a known food source for shorebirds; however, to the best of our knowledge, Haplosporidia have not been previously associated with birds.

Viral diseases play an increasingly important role in the health of aquatic birds, as has been suggested in previous studies^38–40^. Particularly, herpesviruses have been previously associated with respiratory and enteric disease and mortality among seabirds and waterfowl^41–43^. In turn, even though the natural host for ostreid herpesvirus type-1 (OsHV-1) are oysters, Bookelaar et al.^44^ recently determined the potential for *C. edule* to become infected with and to act as an alternate host of OsHV-1 μVar. The presence of herpesviruses in the sediment has been also described. Honjo et al.^45^ reported for the first time Cyprinid herpesvirus 3 (CyHV-3) DNA in sediments of natural lakes and ponds suggesting that sediment could act as a reservoir for CyHV-3 in natural freshwater environments. Subsequently, Evans et al.^46^ detected OsHV-1 in the mangrove sediments from an estuary in Australia.

Therefore, the environmental reservoirs along with the hosts may largely influence the ecology of those pathogen groups by favouring their survival and dispersion in the environment and also serving as a vector for their associated disease. Free-living birds might be involved in the carriage of microbial pathogens not only as biological carriers (the pathogen multiplies in the avian body) but also as mechanical carriers (the pathogen does not multiply in or on the bird)^41^. The pathogen can be located on the surface of the bird’s body or pass through the digestive tract, being viable when excreted^41^. Likewise, birds are hosts for many parasites that sometimes serve as vectors of infectious agents^41^. Birds, including waterfowl, can be hyperparasitized by microsporidians that infect their parasites^47,48^. Hyperparasitism by microsporidia might influence trophic relationships between invertebrate hosts such as *C. edule* and vertebrate hosts such as birds, releasing the secondary host from metacercarial infections^49^. Therefore, birds may play an important role as a reservoir of infection and/or incidental carrier influencing pathogen transmission, dispersal, and disease dissemination. The potential for transport and dissemination of certain pathogenic microorganisms by free-living birds is of concern and is the subject of increased vigilance. For instance, the roles of wild birds in the spread of avian flu and tick-borne viruses are well recognised^50,51^.

Parasite-inclusive food web studies have so far mainly considered macroparasites; however, the large diversity of microparasites and pathogens in mudflat ecosystems remains to be fully explored^1^. Even though it is well-known that infectious agents may incur costs for their avian hosts at an individual level^8^, the trophic transmission of microbial and parasitic infections in aquatic birds and their influence on disease dynamics and dissemination are difficult to establish. Further complications arise from the fact that some host groups such as birds are often transient components of the local food web that can carry parasites and pathogens acquired elsewhere^1^. Polymerase chain (PCR)-based techniques for detecting prey remains in the gut, faeces and regurgitates of predators can be applied to study complex trophic interactions in the field; however, their application is still a challenge. The lack of sensitivity, short post-ingestion detection periods and cross-amplification problems are the major difficulties for detecting degraded, semi-digested DNA^52^. Nevertheless, following stringent contamination control strategies when collecting samples, prompt preservation of those samples and assay optimization can prevent these problems^53–56^.

The presence of the *Vibrio* genus in *C. edule* has been confirmed on the eastern and southern Irish coasts^57^. Likewise, there have been previous records of infected *C. edule* populations with haplosporidian protists such as the genus *Minchinia*^34,35^, or with ostreid herpesvirus type-1 (OsHV-1)^44^, as well as records of microsporidian species parasitizing digeneans that infect cockle populations in those areas^13,49^. The objective of this study was to assess if pathogens associated with *C. edule* (*Vibrio* spp., Haplosporidia spp., ostreid herpesvirus-1 microVar, Microsporidia spp.) could be detected seasonally in the sediment and the faeces of shorebird species at sample sites along the Irish and Celtic Seas. This study used PCR-based techniques adopting strict contamination control strategies and optimisation of the storage, extraction, and purification of the DNA from bird faecal droppings, sediment, and cockles. Shorebird species present were recorded while foraging, with particular attention to cockle consumption. The observational data from shorebirds was assessed in relation to *C. edule* DNA presence and pathogen detection in faecal samples. The spatial and seasonal influence in the detection of the infectious agents in cockles, shorebird faecal samples as well as sediment was also evaluated. Findings will provide an insight into the pathogen community present in *C. edule*, shorebird populations and sediment, establishing the potential of the different pathogen groups to persist in these three natural compartments and be trophically transmitted from *C. edule* to shorebirds. This study highlights the role of the shorebirds as carriers of infectious agents as well as the role of the sediment in the pathogen persistence.

## Results

### Bird species and observational studies

A detailed list of the bird species identified as well as the behaviour observed in the recording is provided in Supplementary Table S1, likewise, their diet and status are described in Supplementary Table S2.

Overall, the bird presence on the sampled cockle beds, in the intertidal and subtidal areas, was constant at the sample sites and throughout the year (Supplementary Table S1). The only exception was in spring 2019 in Annagassan, where there was a clear disturbance in the shorebird community due to a flying drone in the area. A variety of waders (Supplementary Table S1)—oystercatchers *Haematopus ostralegus*, curlews *Numenius arquata*, bar-tailed godwits *Limosa lapponica,* black-tailed godwits *Limosa limosa,* common redshanks *Tringa totanus*, red knot *Calidris canutus* and dunlins *Calidris alpina—*that feed on bivalves, including cockles, were identified in the recordings along with several species of seagulls—*Larus canus, Chroicocephalus ridibundus, Larus marinus, Larus melanocephalus—*and hooded crows (*Corvus cornix*), both groups with a generalist diet but that can feed on bivalves in coastal areas (Supplementary Table S2). Most waders observed were actively foraging and feeding (Table 1; Supplementary Table S1). Some recording was done of birds, especially oystercatchers, digging out shellfish from the sediment and opening them with the beak. Gulls, in turn, were usually loafing, preening, and passively foraging and feeding (Table 1; Supplementary Table S1).

**Table 1:** The most abundant shorebird species, number, location and behaviour observed at each sample site by season, compiling the main flocks observed in the field.

Sites	Sampling dates	Tidal zonation	Bird species	Number of individuals	Behaviour*
Ringaskiddy	Spring 2018	Intertidal	Common gulls (*Larus canus*)	5	O
	Autumn 2018	Intertidal	Oystercatchers (*Haematopus ostralegus*)	3	F
	Winter 2018/19	Intertidal	Oystercatchers (*Haematopus ostralegus*)	5	F
	Spring 2019	Intertidal	Common gulls (*Larus canus*)	2	O
			Great black-backed gulls (*Larus marinus*)	2	
Cuskinny	Spring 2018	Intertidal	Hooded crows (*Corvus cornix*)	Flock (~ 10)	F/O
	Autumn 2018	Intertidal	Black-headed gull (*Chroicocephalus ridibundus*)	Flock (40–50)	F/O
	Winter 2018/19	Intertidal	Common gulls (*Larus canus*)	Flock (20–30)	F/O
			Hooded crows (*Corvus cornix*)	Flock (10–20)	
	Spring 2019	Intertidal	Black-headed gull (*Chroicocephalus ridibundus*)	Flock (30–50)	F/O
Youghal	Summer 2018	Intertidal	Red knot (*Calidris canutus*)	Flocks (10–20)	F
	Autumn 2018	Intertidal / subtidal	Common gulls (*Larus canus*)	4	O
	Winter 2018/19	Intertidal / subtidal	Oystercatchers (*Haematopus ostralegus*)	4	F
	Spring 2019	Intertidal	Oystercatchers (*Haematopus ostralegus*)	3	F
Dungarvan	Summer 2018	Intertidal	Oystercatchers (*Haematopus ostralegus*)	4	F
	Autumn 2018	Intertidal	Black-headed gull (*Chroicocephalus ridibundus*)	Flock (20–30)	F/O
	Winter 2018/19	Intertidal	Common gulls (*Larus canus*)	Flock (20–30)	O
			Great black-backed gulls (*Larus marinus*)	Flock (20–30)	
	Spring 2019	Intertidal	Oystercatchers (*Haematopus ostralegus*)	Flocks (~ 10)	F
Annagassan	Summer 2018	Intertidal	Dunlins (*Calidris alpina*)	Flock (10–20)	F
	Autumn 2018	Intertidal	Oystercatchers (*Haematopus ostralegus*)	Flocks (~ 10)	F
			Common redshank (*Tringa totanus*)	Flocks (~ 10)	
	Winter 2018/19	Intertidal	Oystercatchers (*Haematopus ostralegus*)	Flocks (10–20)	F
			Black-tailed godwits (*Limosa limosa*)	Flocks (10–20)	
	Spring 2019	Supratidal	Raven (*Corvus corax*)	1	F
Cooley	Summer 2018	Intertidal	Oystercatchers (*Haematopus ostralegus*)	Flocks (~ 10)	F
	Autumn 2018	Intertidal	Black-tailed godwits (*Limosa limosa*)	Flock (20–30)	F
	Winter 2018/19	Intertidal	Oystercatchers (*Haematopus ostralegus*)	Flocks (~ 10)	F
	Spring 2019	Intertidal / subtidal	Black-tailed godwits (*Limosa limosa*)	Flock (20–30)	F
			Common gulls (*Larus canus*)	Mixed flock (> 50)	F/O
			Mediterranean gulls (*Larus melanocephalus*)		
			Sandwich terns (*Sterna sandvicensis*)		

(*) Following the descriptions in Lewis and Tierney^58^, shorebird behaviour was classified in two categories: (F) foraging/feeding: the active or passive search of food and feeding; and (O) other: all other behaviours that do not assign to foraging, including roosting, standing, preening, loafing, swimming and others.

The sample sites with lower bird occurrence were Ringaskiddy and Youghal, where a few scattered bird individuals were recorded (mainly *H. ostralegus*, *N. arquata* and *L. canus*), except for a flock of *C. canutus* (10 ≤ 20 individuals) in summer 2018 at Youghal (Table 1; Supplementary Table S1). Bird flocks (> 10 individuals) were common in Cuskinny, Dungarvan, and Dundalk (Annagassan and Cooley) (Table 1; Supplementary Table S1). Large gull flocks (> 20 individuals) were recorded at Cooley and, especially, at Cuskinny and Dungarvan (Table 1; Supplementary Table S1). In turn, wader flocks (10 ≤ 30 individuals), including *H. ostralegus*, *L. limosa*, *T. totanus* and *C. alpina*, were more frequently recorded in Dundalk sites (Annagassan and Cooley) (Table 1; Supplementary Table S1). Gulls were recorded over the year, except for the winter season in Annagassan and Cooley, while waders seemed to be more frequently observed during autumn and winter (Supplementary Table S1), which would correlate with their status as winter visitors to Ireland (Supplementary Table S2).

### Molecular cockle species identification

All the screened cockles were identified as *Cerastoderma edule*, with no presence of *Cerastoderma glaucum* or hybrids.

Among the faecal and sediment samples taken, 61.8% (n = 126/204) of the faecal samples and 63.2% (n = 129/204) of the sediment samples showed good DNA quantity (≥ 1 ng/µl) and high-quality DNA (A260/A280 = 1 ≤ 1.99), while 17.6% (n = 36/204) of the faecal samples and 18.1% (n = 37/204) of the sediment samples showed good DNA quantity (≥ 1 ng/µl) and presence of RNA along with DNA (A260/A280 = 2 ≤ 4).

**Figure 1:**
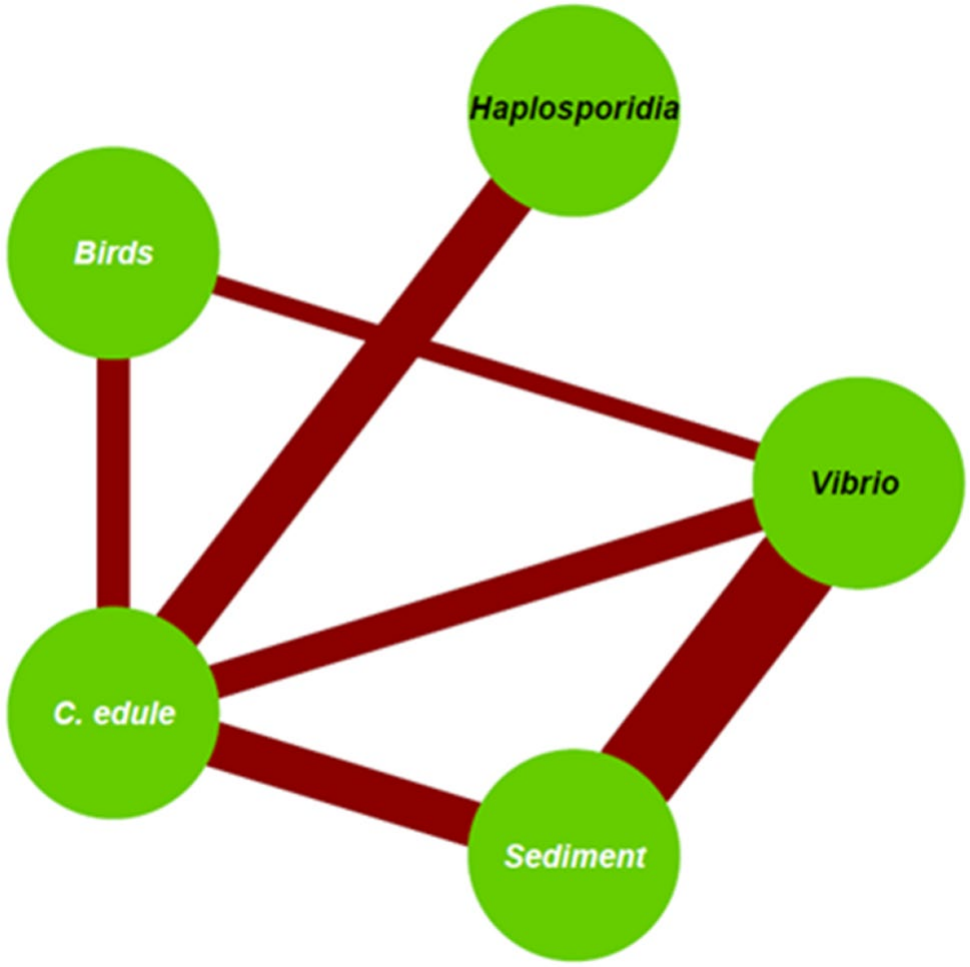
Network association plot displaying the links between the different compartments studied. The black text refers to pathogens and the white text relates to hosts/reservoirs. A solid line represents target DNA presence and line thickness indicates the prevalence (%) of infected *C. edule* and the percentage of positive faecal and sediment samples.

In the seabird faecal samples, 22.5% (n = 46/204) contained *C. edule* DNA (Fig. 1), with 63.0% (n = 29/46) of those samples displaying high quantities of DNA (≥ 1 ng/µl) and high-quality DNA (A260/A280 = 1 ≤ 1.99). In the sediment samples, 33.8% (n = 69/204) contained *C. edule* DNA (Fig. 1), with 56.5% (n = 39/69) of those samples displaying good quantities of DNA (≥ 1 ng/µl) of high-quality (A260/A280 = 1 ≤ 1.99). There was no presence of *C. glaucum* DNA or hybrids in any seabird faecal or sediment samples.

**Figure 2:**
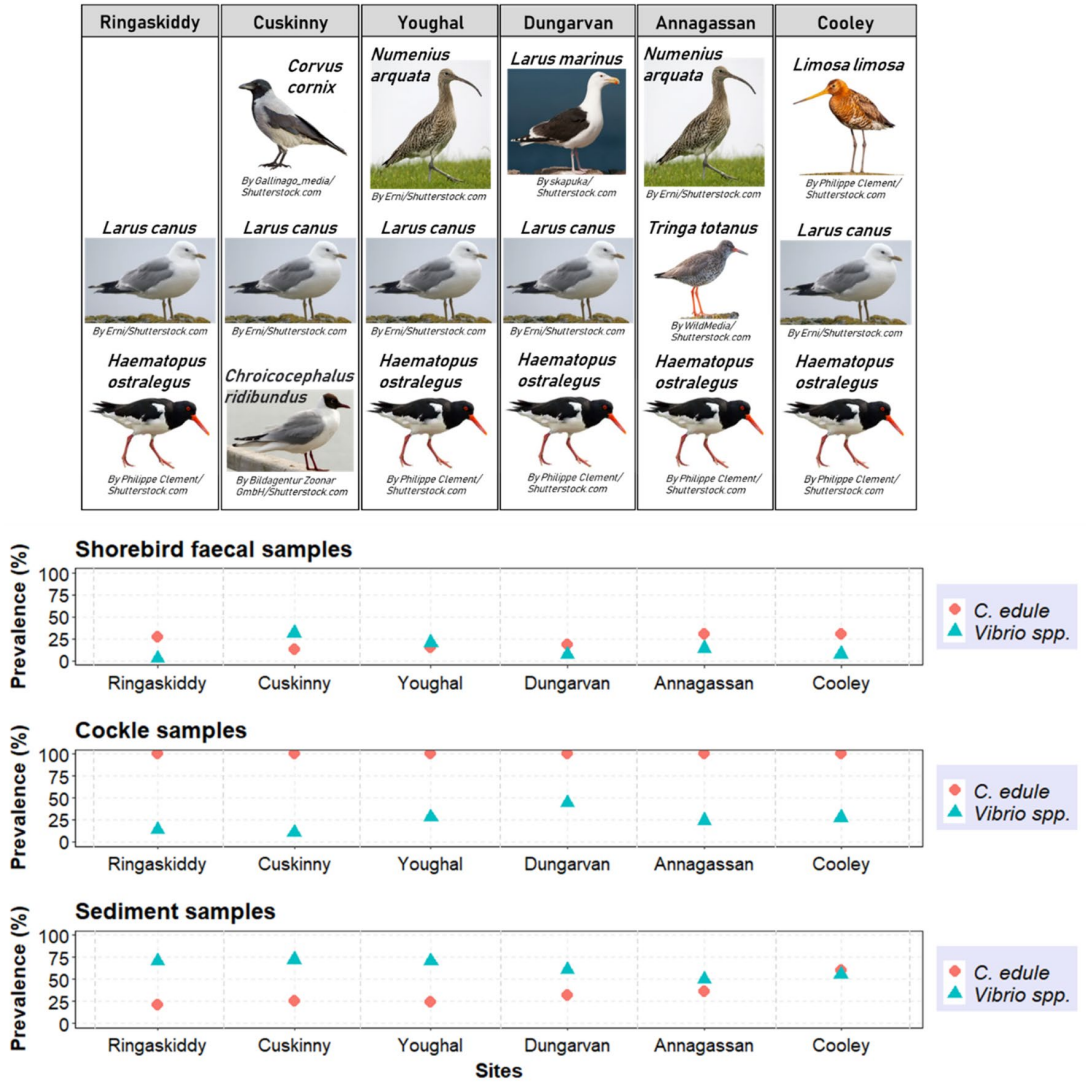
Distribution of *C. edule* DNA compared to the presence of *Vibrio* spp. in shorebird faecal samples, cockles, and sediment samples at each sample site. The most frequently observed shorebird species throughout the seasons at each sample site are represented on the top of the figure (Images used under license from Shutterstock.com).

The highest percentage of *C. edule* DNA detected in faecal samples occurred at Dundalk Bay – Annagassan (30.6%; n = 11/36) and Cooley (30.0%; n = 12/40) –, where the cockle abundance found was high compared to the other sites and where oystercatchers *H. ostralegus*, curlews *N. arquata*, black-tailed godwits *L. limosa*, common redshanks *T. totanus* and dunlins *C. alpina* were observed foraging (Fig. 2)*.* Cuskinny, where gulls were the dominant species recorded and the cockle abundance observed in that area was lower than other sites, was the site with the lowest level of *C. edule* DNA found in faeces (12.5%; n = 4/32) (Fig. 2). Youghal, with a low cockle and bird abundances observed, also showed a low detection of *C. edule* DNA in faeces (14.7%; n = 5/34) (Fig. 2). The lowest *C. edule* DNA detection in the sediment samples was in Ringaskiddy (20.6%; n = 7/34), where a low abundance of cockles and empty cockle shells were found at every sampling, while the highest detection was in the commercial fishery of Dundalk—Annagassan (36.1%; n = 13/36) and Cooley (60.0%; n = 24/40) -, with higher cockle abundances observed (Fig. 2).

**Figure 3:**
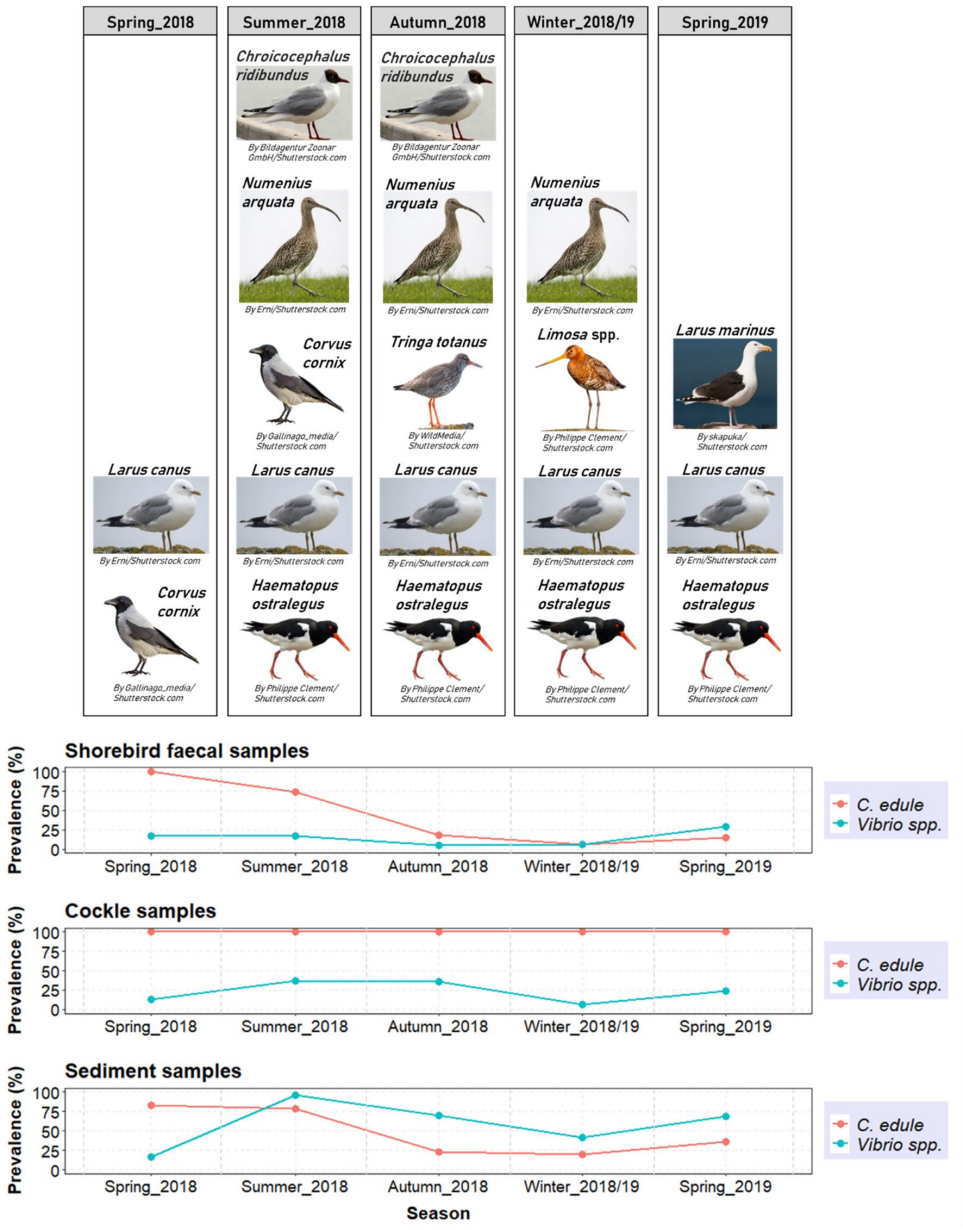
Distribution of *C. edule* DNA compared to the presence of *Vibrio* spp. in shorebird faecal samples, cockles, and sediment samples throughout the seasons. The most frequently observed shorebird species through the sites by each season are represented on the top of the figure (Images used under license from Shutterstock.com).

Seasonal variability in the detection of *C edule* DNA in faecal and sediment samples was observed, with the highest detection of *C edule* DNA during spring 2018 (faeces: 100%, n = 6/6; sediment: 83.3%, n = 5/6) and summer 2018 (faeces: 73.9%, n = 17/23; sediment: 78.3%, n = 18/23), when more screened cockles were found dead or in poor condition (full of sand and bad smell); while the lowest detection was in winter 2018/19 (faeces: 6.7%, n = 4/60; sediment: 20.0%; n = 12/60) (Fig. 3). Flocks of *H. ostralegus, C. alpina* and *C. canutus* (10 ≤ 20 individuals) were observed during summer, while *H. ostralegus*, *T. tetanus*, and *L. limosa* flocks (10 ≤ 30 individuals) along with large flocks of gulls (20 ≤ 50 individuals) and hooded crows (10 ≤ 20 individuals) were observed in autumn and winter (Fig. 3).

### Pathogen screening

During the study two pathogen groups were detected in *C. edule* samples: 37.7% (n = 277/735) tested positive for Haplosporidia, while 25.3% (n = 186/735) tested positive for *Vibrio* spp. (Fig. 1; Table 2). Of the 277 cockles positive for Haplosporidia, 29.6% (n = 82/277) could be distinguished as being infected with *Minchinia tapetis* and 14.8% (n = 41/277) were infected with *Minchinia mercenariae*-like (Table 2). However, other haplosporidian species not targeted by this study could also be present in the *C. edule* samples that were positive in the generic haplosporidian PCR. Of the 186 cockles positive for *Vibrio*, 19.9% (n = 37/186) were determined to be infected with *Vibrio aestuarianus* (Table 2), with highly infected individuals defined as those individuals that showed a C_t_ < 30, since the *V. aestuarianus* positive controls (diluted purified DNA) had a C_t_ = 30. Based on that, 13.5% (n = 5/37) of the individuals determined to be positive for *V. aestuarianus* were classified as heavily infected individuals, while 86.5% (n = 32/37) were classified as having light/low infections. Dungarvan Harbour was the site with the highest presence of *V. aestuarianus* and three out of the five heavily infected individuals were found in that site during summer 2018. Sequencing was conducted with a subsample of the other 80.1% (n = 149/186) of samples that were not positive for *V. aestuarianus*, results listed below (Table 3). No ostreid herpesvirus-1 microVar nor Microsporidia spp. were detected in any of the *C. edule* screened during the study (Table 2).

**Table 2:** Total numbers of positive cases (%, positive individuals/screened individuals) for the screening done for the target pathogen species in cockles, seabird faecal samples and sediment samples.

Pathogen species	Cockle samples	Seabird faecal samples	Sediment samples
Haplosporidia	**37.7% (277/735)**	0% (0/84)	0% (0/84)
*Minchinia tapetis*	**29.6% (82/277)**	0% (0/84)	0% (0/84)
*Minchinia mercenariae*-like	**14.8% (41/277)**	0% (0/84)	0% (0/84)
Ostreid herpesvirus type-1 and variants	0% (0/735)	0% (0/113)	0% (0/113)
Microsporidia sp.1	0% (0/735)	0% (0/58)	0% (0/58)
Microsporidia sp.2	0% (0/735)	0% (0/58)	0% (0/58)
*Vibrio* spp.	**25.3% (186/735)**	**13.7% (28/204)**	**62.7% (128/204)**
*Vibrio aestuarianus*	**19.9% (37/186)**	0% (0/204)	**1.6% (2/204)**

**Table 3:** Description of the Blast results obtained from the sequenced DNA of cockles, seabird faeces and sediment samples using generic *Vibrio* primers.

Sample type	Sample site	Season	Species identification	Percent Identity	Query Cover	Query Length (bp)
*Cockles*	Youghal Dungarvan Cooley	Summer 2018	*Vibrio spp.* among them: Vibrio splendidus* and V. kanaloae*	92.59–98.58%	78–83%	82–179
*Cockles*	Annagassan Annagassan	Summer 2018	*Vibrio spp*. most likely V. splendidus*	97.84–97.92%	88–91%	154–155
*Cockles*	Annagassan	Summer 2018	*Vibrio spp.* most likely *V. aestuarianus* or *V. mediterranei*	98.01%	94%	158
*Cockles*	Cuskinny Dungarvan	Autumn 2018	*Vibrio spp.*	94.23–94.29%	67–73%	52–69
*Sediment*	Cooley Dungarvan	Summer 2018	*Vibrio spp.*	95.31–95.83%	85–93%	74–75
*Sediment*	Ringaskiddy	Autumn 2018	*Vibrio splendidus*	92.73%	85%	62
*Sediment*	Ringaskiddy Ringaskiddy	Autumn 2018 Winter 2018	*Vibrio sp*. most likely V. splendidus*	95.65–98.51%	60%	110–114
*Sediment*	Youghal	Summer 2018	*Vibrio sp.* most likely V. kanaloae*	94.37%	30%	222
*Shorebird faeces*	Dungarvan	Spring 2019	*Vibrio sp*. most likely V. splendidus*	98.67%	86%	87
*Shorebird faeces*	Cooley	Summer 2018	*Grimontia hollisae (previously classified as Vibrio hollisae)*	97.14%	50%	138
*Shorebird faeces*	Youghal	Spring 2019	*Vibrionaceae bacterium*	100%	80%	57
*Shorebird faeces*	Annagassan	Autumn 2018	Enterobacter/Kiebsiella spp.	96.43%	73%	75
*Shorebird faeces*	Cuskinny	Autumn 2018	*Enterobacterales*	98.48%	44%	146

(*) Same strains of *Vibrio* spp. were identified in cockle samples and sediment/faecal samples.

Of the various pathogen groups screened for, only vibrios were found in both bird faecal samples and sediment, apart from in *C. edule* samples (Fig. 1). *Vibrio* spp. DNA was detected in 13.7% of the seabird faeces (n = 28/204) and 62.7% (n = 128/204) of the sediment samples (Table 2). 64.3% (n = 18/28) of those faecal samples and 66.4% (n = 85/128) of those sediment samples displayed good quantities of DNA (≥ 1 ng/µl) and high-quality DNA (A260/A280 = 1 ≤ 1.99). The PCR positive samples for generic *Vibrio* in the three screened sample types were defined as *Vibrio* spp. for analysis purposes. However, specific screening of *V. aestuarianus* was conducted by qPCR and sequencing done with subsamples of the *C. edule*, faecal and sediment samples revealed other *Vibrio* species present during the study but not quantified.

*V. aestuarianus* was absent in the faecal samples, and it was only detected in two of the sediment samples (1.6%, n = 2/204), in spring 2018 at Cuskinny and in summer 2018 at Dungarvan (Table 2), and both were classified as having low bacteria loading. The strain of *V. aestuarianus* detected in cockles and sediment was confirmed to be the same (MK307696.1, strain 15_075_1T2), which has been recently confirmed to be pathogenic to the common cockles *C. edule*^59^. Other species of *Vibrio*, known as important aetiological agents of diseases affecting all life stages of shellfish^18,21^, were also identified: *Vibrio splendidus* and *Vibrio kanaloae*. It was confirmed that three strains of *V. splendidus* identified by direct Sanger sequencing were the same in the three sample types (Table 3). The sequences obtained from the three samples types, with a query length between 87–155 bp, showed 60–91% query coverage and 92.6–98.7% percent identity to *V. splendidus* strains deposited by Gao (2020) (MT445179.1, strain FS1; MT445177.1, strain CT1) and by Landreau et al. (2020) (MT345091.1, strain D0-B01). Some of these sequences obtained from *C. edule* and bird faecal samples came from the same sampling site, Dungarvan (Table 3). However, the identified sequences from the sediment are from Ringaskiddy. Likewise, the *V. kanaloae* strain CAIM 485 was found by sequencing in both cockle and sediment samples (Table 3). The cockle samples, with a query length between 82–179 bp, showed 78–83% query coverage and 92.6–98.6% percent identity to *V. kanaloae* deposited by Sun (2020) (MT759943.1); while the sequence from the sediment sample, with a query length of 222 bp, showed 30% query coverage and 94.4% percent identity to *V. kanaloae* deposited by Sun (2020) (MT759943.1). Nevertheless, it was not considered a robust outcome due to the low query cover (30%) in the sequenced sediment sample. For two of the sequenced cockle samples, it was not possible to generate sequences that were long enough to identify species.

The prevalence of *Vibrio* spp. displayed significant spatial variability (p(Chisq) < 0.05) in both *C. edule* and bird faecal samples (Fig. 2), although, no significant correlation (p(τ) > 0.05) in the prevalence of *Vibrio* spp. was established between cockles and shorebird compartments. Ringaskiddy displayed the lowest prevalence of *Vibrio* spp. (2.9%; n = 1/34) in the bird faecal samples, but also showed a low prevalence of *Vibrio* spp. in *C. edule* samples (13.7%; n = 16/117) (Fig. 2). In turn, Youghal, Annagassan and Cooley showed intermediate values in the prevalence of *Vibrio* spp. detected in *C. edule* samples and bird faecal samples (Fig. 2). Cuskinny was the site showing the lowest prevalence of *Vibrio* spp. in cockle samples (10.7%; n = 15/140), while Dungarvan presented the highest prevalence (44.4%; n = 75/169) (Fig. 2). In contrast, the highest prevalence of *Vibrio* spp. detected in faecal samples was in Cuskinny (31.3%; n = 10/32), with the lowest level of *C. edule* DNA found in the faeces of that area (12.5%; n = 4/32) (Fig. 2); while Dungarvan was the second site with the lowest prevalence of *Vibrio* spp. in faecal samples (7.1%; n = 2/28) and a low level of *C. edule* DNA found in the faeces (17.9%; n = 5/28) (Fig. 2). No significant correlation (p(τ) > 0.05) was established between the presence of *C. edule* DNA and *Vibrio* DNA detected within the shorebird compartment.

The presence of *Vibrio* spp. in sediment samples overpassed the prevalence of *Vibrio* spp. in bird faecal samples and to a lesser extent in *C. edule* samples (Fig. 2). The examination of collinearity in the prevalence of *Vibrio* spp. between the different compartments—sediment, cockles, and shorebirds – revealed a correlation (τ > 0.3) between sediment and cockles.

In sediments samples, no significant differences in *Vibrio* presence between sample sites were displayed (p(Chisq) > 0.05). The occurrence of *Vibrio* spp. remained fairly high through the sites, regardless of the different levels of *C. edule* DNA found in the sites—no significant correlation (p(τ) > 0.05) was established between the presence of *C. edule* DNA and *Vibrio* DNA detected within the sediment compartment—(Fig. 2).

Significant seasonal variability (p(Chisq) < 0.01) in the occurrence of *Vibrio* spp. was exhibited in the three studied compartments—sediment, cockles, and shorebirds-. The highest presence of *Vibrio* spp. in *C. edule*, bird faecal samples and sediment samples, was during the warmer months and declined during the winter (Fig. 3). The presence of *Vibrio* spp., therefore, followed the same seasonal trend as the occurrence of *C. edule* DNA in both faeces and sediment samples (Fig. 3), although the correlation between *C. edule* DNA and *Vibrio* DNA detected in those compartments was not statistically significant (p(τ) > 0.05). 28.6% (n = 8/28) of the faecal samples that were positive for *Vibrio* spp. also contained *C. edule* DNA and all of them were from spring and summer. It was also noticeable that in spring 2019 the prevalence of infection was higher in the three compartments compared to spring 2018 (Fig. 3) when the only samples available were from Ringaskiddy and Cuskinny.

## Discussion

This study demonstrates connectivity between the presence of vibrios in the marine environment and the different compartments that they can be detected in, i.e. sediment, invertebrates, and vertebrates. The presence of *Vibrio* spp. was confirmed in sediment samples, that may act as a reservoir or sink for such bacteria; in sediment-dwellers *C. edule*, where some may be pathogenic; and in shorebirds, infected via the consumption of cockles, resulting in transmission of these bacteria via faeces and the further repopulating of the sediment by these faecal bacteria. Unlike *Vibrio* spp., haplosporidians, including *Minchinia* spp., were found in *C. edule* samples but no transmission to shorebirds was detected. Haplosporidia DNA was also absent in the sediment. The lack of detection of haplosporidians supports the effectiveness of the *Vibrio* pathogen group to be transferred through the bird digestive system and persist in the environment. This finding may provide new insight into the life cycle of haplosporidians, which is still not fully known, and its capacity of persistence outside the host. However. further research in the infective stages of these pathogens is needed.

Two *Vibrio* species were identified that were of interest due to their pathogenicity and their major role in bivalve disease outbreaks^20,59–62^; *Vibrio splendidus*, known as an important aetiological agent of diseases affecting *Crassostrea gigas* aquaculture^60,61^, and *Vibrio aestuarianus*, whose harmful effect has been reported on *C. edule*^59^ and *C. gigas*^63^*.* For the first time, to the best of our knowledge, identical strains of *V. splendidus* have been reported in *C. edule,* faecal samples of wild shorebirds and sediment. *V. aestuarianus* was detected in *C. edule* through the sample sites and in sediment from Cuskinny and Dungarvan, but it was absent in the faecal samples. A potential explanation for this absence is that the bacterial load in cockles was not high enough to be transmitted to the birds during foraging or to be detected due to the low levels present.

*V. splendidus* detection in bird faeces further supports the carrier role that migratory birds play in the dissemination of *Vibrio* species, such as in *V. cholerae*^64–67^, *V. parahaemolyticus, V. vulnificus* and *V. mimicus*^68–70^. It is well-known that the dispersal of *V. cholerae* by aquatic birds may be attributable to their direct predation of fish^71–73^, chironomids and copepods^64^. Recently, Fu et al.^70^ demonstrated that the predation of molluscs and zooplankton by migratory birds played an essential role in the dissemination of *V. parahaemolyticus* and *V. mimicus* in the estuary of the Liaohe River in China. However, the transmission of *Vibrio* species less relevant for humans had been neglected and very little is known about the role that migratory birds play in the spread of these pathogens. Our findings, therefore, provided the first evidence of possible transmission of *V. splendidus* to birds through consumption of infected cockles, as was supported by the presence of *C. edule* DNA and *Vibrio* DNA in the same bird faecal samples. This was further supported by the observations of waders, mainly oystercatchers *H. ostralegus*, known to feed on bivalves including cockles, at the sample sites. Cockle availability is a key resource supporting many overwintering wader populations such as oystercatchers^5^. The observational data collected also revealed that the presence of waders was larger at the sample sites with higher cockle abundances observed during the sampling. Previous studies have demonstrated a positive correlation between shorebird abundance and invertebrate prey densities, especially when patterns are examined across large spatial scales (e.g., encompassing entire estuaries)^74–76^. Moreover, the identified strains of *V. splendidus* in *C. edule* and bird faecal samples came from the same sample site, Dungarvan, with the highest prevalence of *Vibrio* spp. in *C. edule* samples.

The spatial variability in the *Vibrio* infection established in *C. edule* and bird faecal samples added evidence to the influence that cockle consumption may have had on the *Vibrio* prevalence detected in shorebird faecal samples. Wader flocks were common in Dundalk (Annagassan and Cooley) with the highest presence of cockles observed in the field and the highest densities reported in the literature (Shellfish Stocks and Fisheries Review, Marine Institute and BIM, 2017). Both sites showed the highest presence of *C. edule* DNA in the bird faeces, suggesting the waders are feeding on the cockles of those areas. In turn, Annagassan and Cooley showed intermediate values in the prevalence of *Vibrio* spp. detected in both *C. edule* samples and bird faecal samples. Despite the more frequent observation of waders in the sites with easier access to cockles, the correlation in *Vibrio* prevalence between *C. edule* and shorebird compartments was not significant. The strongest discrepancy in the prevalence of *Vibrio* spp. detected between the *C. edule* samples and the faecal samples was found at Cuskinny and Dungarvan. Dungarvan, with the highest prevalence of *Vibrio* spp. and *V. aestuarianus* in *C. edule*, showed a low prevalence of *Vibrio* spp. in bird faecal samples, probably due to the predominant presence of gulls, with a generalist diet, with respect to waders. Cuskinny exhibited the lowest prevalence of *Vibrio* spp. in cockle samples and the highest prevalence of *Vibrio* spp. in faecal samples, being also the gulls the dominant birds present. Therefore, it may reflect a lower consumption of cockles by gulls. This is consistent with the lower presence of *C. edule* DNA found in bird faecal samples at both sites. In the case of Cuskinny, the high prevalence of *Vibrio* spp. in faecal samples may be due to another source of *Vibrio* rather than the consumption of cockles.

Seasonal variability in the *Vibrio* infection was confirmed in *C. edule* and bird faecal samples and may also be related to the cockle consumption by shorebirds. Prevalence of *Vibrio* spp. increased in *C. edule* and faecal samples during the warmer months, probably due to the higher temperature. Temperature is likely the most important driver of the overall change in *Vibrio* abundance in temperate coastal waters^27^. Temperature promotes the proliferation of bacteria such as *Vibrio*, which grow preferentially in warm waters^26,27^. The high seawater temperature may also have an important impact on the physiological state of bivalve hosts, which makes them more susceptible to infection during the warm period of the year^77^. Consequently, infected *C. edule* may have been more accessible for shorebirds at that time of the year, when more screened cockles were found in poor condition. This higher susceptibility of cockles may have also promoted its opportunistic consumption by generalist feeders such as gulls and hooded crows, as suggested by the higher occurrence of *C. edule* DNA detected during the warmer months in the bird faecal samples. Although no significant correlation was established between the presence of *C. edule* DNA and *Vibrio* DNA in faecal samples, *Vibrio* prevalence followed the same seasonal trend as the occurrence of *C. edule* DNA in bird faeces. It was also noticeable that in spring 2019 the prevalence of infection was higher in *C. edule* and faecal samples compared to spring 2018. This might be because the only samples available in spring 2018 were from Ringaskiddy and Cuskinny, where the prevalence of *Vibrio* spp. was low in *C. edule* samples. Besides, in Cuskinny, gulls, which are not exclusively dependent on cockles for their feeding, were most frequently observed.

As expected, due to the infaunal lifestyle, *C. edule* DNA was also detected in sediment samples, with higher frequency than in the faecal samples. The detection of *C. edule* DNA in the sediment samples was higher at the sites with higher cockle abundances observed, i.e. Dundalk (Annagassan and Cooley). The detection of *C. edule* DNA in Ringaskiddy and Youghal was the lowest, as they were the areas where more effort was required to find enough cockles for the study. Empty cockle shells were observed at Ringaskiddy at every sampling, while Youghal is an extremely muddy shore, which makes it less suitable for cockles that prefer a sand-mud mixture best for cockle growth^5^. A significant correlation between the occurrence of *Vibrio* spp. in cockles and sediment was established, confirming the link between these two compartments. In sediment samples, the total frequency of *Vibrio* spp. detected was higher than in shorebird faeces and *C. edule* samples. Previous studies have shown that attachment to surfaces, such as the exoskeleton of chitin as well as sediment, is an integral part of the aquatic lifestyle of many vibrios, representing a successful survival mechanism^27,28,78,79^. Vezzulli et al.^27^ observed that both *V. aestuarianus* and *V. splendidus* maintained viability and culturability for longer times in the sediment than in seawater in laboratory experiments, suggesting that this compartment may represent a suitable niche for their persistence in the environment. Accordingly, Johnson et al.^11^ observed a protective effect in sediment compared with oysters and seawater, for *V. parahaemolyticus* and *V. vulnificus*. Freitas et al.^80^ recently demonstrated that *V. parahaemolyticus* cells attach to underwater surfaces, such as sediment, and/or associate with various species of shellfish and zooplankton as part of their life cycle. In our study, no significant spatial variability was detected in the presence of *Vibrio* spp. in sediment samples. *Vibrio* spp. frequency remained consistently high through the sample sites regardless of the different levels of *C. edule* DNA detected, with no correlation established between the *C. edule* DNA and *Vibrio* DNA found in the sediment. The findings highlight the role of the sediment as a major reservoir for *Vibrio* spp.

*Vibrio* spp. showed a lower prevalence during winter in both *C. edule* and faecal samples, however, *Vibrio* spp. frequency remained higher in the sediment. Accordingly, Vezzulli et al.^81^ identified the sediment as an environmental reservoir for *Vibrio*, where the bacteria can find a favourable environment for overwintering. This is also probably linked to the fact that sediment provides biotic and abiotic surfaces useful for bacterial biofilm development; moreover, the concentration of organic matter in this compartment is higher than in the overlying water column (10,000–100,000 fold-higher in natural conditions)^81^.

The strains of *V. splendidus* found in the sediment were the same as the ones in *C. edule* and bird faecal samples, although the strains of *V. splendidus* in sediment were identified in a different cockle bed, as well as the same strain of *V. aestuarianus* was detected in *C. edule* and sediment samples. In waterborne infections, in fact, the pathogenic agent can be shed by infected migrating birds, resulting in contamination of water with faeces or other corporal discharges^41^. Subsequently, molluscs inhabiting the area may get infected by filtration of the water column with the pathogenic agent in it^70^. The presence of pathogens in the sediment may also have an impact on the infaunal community within the sediment, which influence ecosystem services as bioturbation, nutrient recycling or carbon sequestration^5^. The presence of the same vibrios in the three analysed compartments expose, therefore, the intricate links of the marine trophic webs and the influence of the environment on them.

The findings obtained from this research provide new insight into the parasite-inclusive food web studies of mudflat ecosystems, targeting microparasites and pathogens that have received much less attention in this respect. The observed differences in the transmission of the Haplosporidia and the *Vibrio* spp. analysed indicates that not all pathogen groups are transmitted via food webs and that certain pathogens such as bacteria can be ingested and excreted with their DNA integrity intact thus indicating viability. The findings shed light on the potential transmission of *V. splendidus* associated with bivalves to shorebirds, via cockle consumption; as it has been previously reported with oysters^67^ and fish^72^, whose intake transmits *V. cholerae* to aquatic birds. Our findings add evidence to the significant role migratory birds may have as carriers of infectious agents such as bacteria, enabling their transport and dispersal not only to short-distance, given the efficient digestion and short food retention time in shorebirds, but also to long-distance if the infection in shorebirds occurs^82^. Further examination of regurgitated pellets and faecal droppings, as well as the analysis of gut contents of shorebirds, would be necessary to definitively confirm the subsequent trophic transmission and the infection success and impacts this pathogen can have on shorebird populations. Likewise, our findings further support the role of sediment as a *Vibrio* reservoir. The analysis of other compartments in the field, such as seawater, cohabiting bivalves, etc., and the factors affecting the survival of the infectious agents, would be of interest for future research to provide a complete picture of the infection sources and the potential connectivity between them.

## Methods

### Sample sites

**Figure 4:**
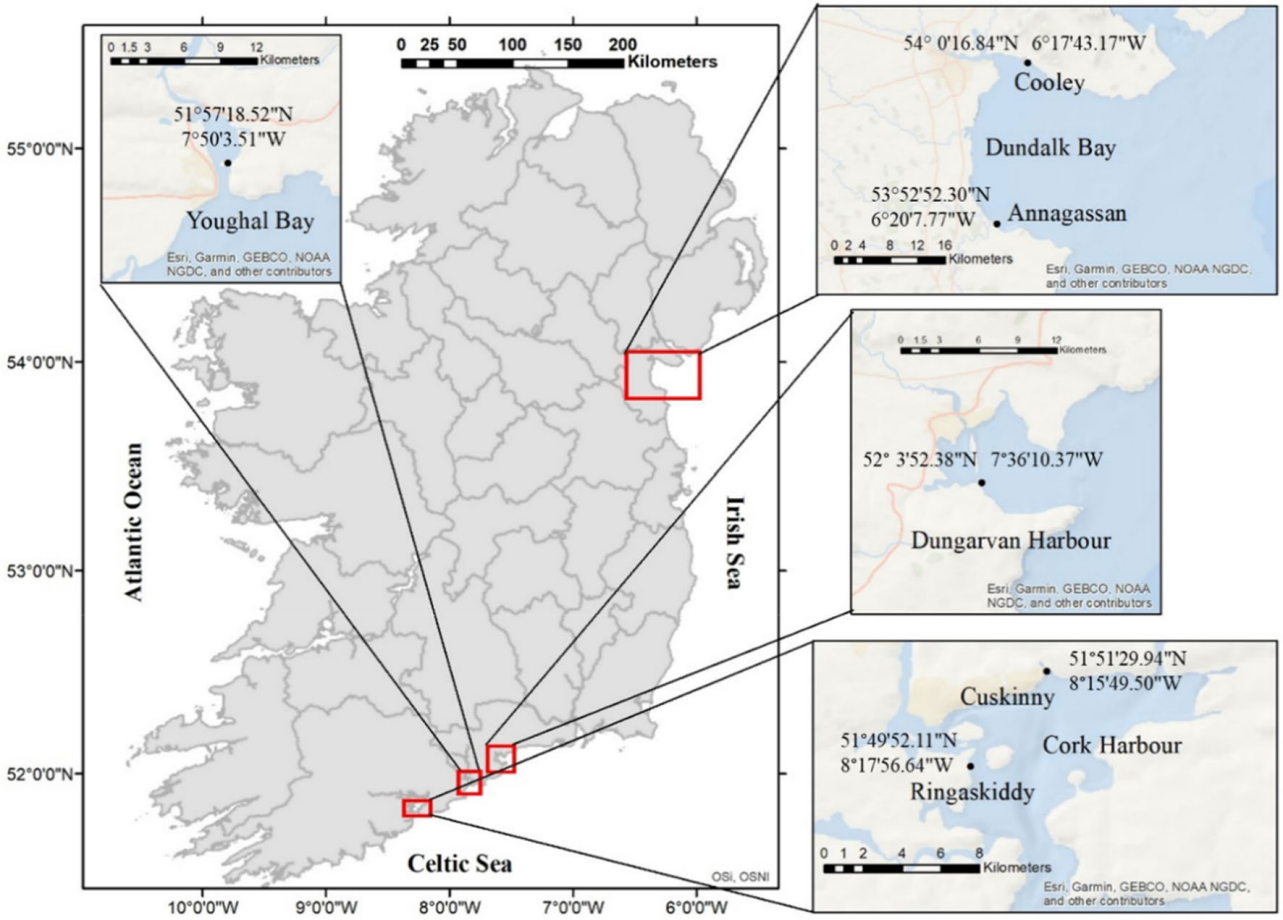
Map of Ireland highlighting the sample sites with coordinates (ArcGIS Desktop 10.5.1; www.esri.com).

Cockles, faecal samples and sediment samples were collected seasonally from April 2018 to April 2019 at Cork Harbour (Ringaskiddy and Cuskinny) on the south coast (Celtic Sea) of Ireland, and from July 2018 to April 2019 at Youghal Bay and Dungarvan Harbour, the latter the site of extensive farming of Pacific oysters (*Crassostrea gigas*), on the south coast (Celtic Sea), as well as at the commercial fishery at Dundalk Bay (Annagassan and Cooley) on the northeast coast (Irish Sea) (Fig. 4). The six locations are bays with intertidal sand and mudflats, where a variety of bivalve species inhabit. They are Special Protection Areas (SPAs) under the E.U. Birds and Habitats Directives, providing good quality feeding areas and roost sites for an excellent diversity of waterfowl species (www.npws.ie/protected-sites/spa). Moreover, Cuskinny is classified as Marsh Nature Reserve and Dundalk Bay is a classified Bivalve Mollusc Production Area and it has supported a commercial dredge cockle (*Cerastoderma edule*) fishery since 2001^83^.

Although the cockle densities in situ were not measured, observations of cockle abundances during the samplings were in accordance with the reference densities previously recorded in those sites. *C. edule* densities in the selected sites varied from higher densities, 9.33 ± 3.5 ind/m^2^, in Dundalk Bay (Shellfish Stocks and Fisheries Review, Marine Institute and BIM, 2017) to lower densities in sites that are not fished: 1.6 ± 2.1 ind/m^2^ in Youghal Bay; 0.8 ± 1.7 ind/m^2^ in Cork Harbour; and 0.4 ± 1.3 ind/m^2^ in Dungarvan Harbour^13^.

### Bird observations

Video recording of shorebirds while foraging at low tide in the study areas was carried out seasonally from April 2018 to April 2019, following the specifications of Martins et al.^84^. Recordings were carried out with a digital camcorder (CX405B, Sony) with a 30× optical zoom. The video recording was done prior to sampling to avoid disturbance. The average duration of the films were 5 to 10 min, each one taken from a different perspective of the bay while filming the main bird aggrupations. Therefore, it is highly unlikely that pseudoreplication (i.e., filming the same individual more than once) affected our dataset and conclusions to any significant extent. The recording was done in the aerially exposed cockle bed, where the faecal samples and the sediment samples were collected at low tide. The viewing of the videos was carried out to list the bird species, their number and behaviour i.e., foraging and feeding or not, at the time of the filming. Following the descriptions in Lewis and Tierney^58^, shorebird behaviour was classified in two categories: (1) foraging/feeding: the active or passive search of food and feeding; and (2) other: this category was used for all other behaviours that do not assign to foraging, including roosting, standing, preening, loafing, swimming and others. Prey identity was not examined. However, in some cases, analysis of video recordings allowed the distinction of the consumption of bivalves, even though identification of specific species could not be done even with the best quality recordings.

### Sample types

Cockles were collected by hand raking from the intertidal area of each site at spring tide (0.5–1 m) and seasonal intervals. The sample size, outlined in Table 4, was dependant on the available abundance at each site and season. Cockles were kept at ambient temperature during the fieldwork and were processed either on the same day or were held overnight at 4 °C and were processed the next day.

**Table 4:** Number of collected cockles (C.), bird faecal samples (F.) and sediment samples (S.) by site and season (Sp.18: spring 2018; Sum.18: summer 2018; Aut.18: autumn 2018; Wint.18/19: winter 2018/19; Sp.19: spring 2019).

Dates	Cork Harbour—Ringaskiddy			Cork Harbour—Cuskinny			Youghal Bay			Dungarvan Harbour			Dundalk Bay – Annagassan			Dundalk Bay -Cooley		
	***C***	***F***	***S***	***C***	***F***	***S***	***C***	***F***	***S***	***C***	***F***	***S***	***C***	***F***	***S***	***C***	***F***	***S***
Sp. 18	29	4	4	31	2	2	–	-	–	–	–	–	–	–	–	–	–	–
Sum. 18	12	–	–	27	–	–	11	4	4	51	3	3	43	6	6	17	10	10
Aut. 18	31	10	10	29	10	10	16	10	10	58	10	10	30	10	10	30	10	10
Win. 18/19	15	10	10	23	10	10	30	10	10	30	10	10	30	10	10	30	10	10
Sp. 19	30	10	10	30	10	10	12	10	10	30	5	5	30	10	10	30	10	10
Totals	117	34	34	140	32	32	69	34	34	169	28	28	133	36	36	107	40	40

(–) No samples collected.

Sediment and seabird faecal samples were collected as near as possible to the cockle collection area (Table 4). As food retention time in the digestive tracts of birds is short—for instance, for red knots (*Calidris canutus*) it is 20–50 min^82^ -, the collection of samples was done after 30 min from the start of the video recording to ensure that the bird faeces collected were composed of food remains found and digested by the shorebirds feeding on prey from that foraging site^84^. Collection of samples was conducted following stringent contamination control strategies, such as using sterilised material and avoiding taking any substrate in contact with the faecal matter. One sterilised swab was used to collect a small amount (around 5 mm^3^) of the bird faecal waste and a second swab was used to collect the same amount of sediment nearby to the stool. Each sample was transferred into an individually labelled 2 ml sterile Eppendorf tube.

Wherever possible, faeces were collected fresh to minimize further enzymatic action and, consequently, degradation of the DNA, although freshness is not always easy to determine in the field^52^. Preservation of faecal samples has mainly been by freezing in a range from −20 °C to −80 °C^53–56^. Seabird faeces and the sediment samples were stored in an insulated bag with iced packs during the fieldwork and, once in the lab, they were frozen at −80 °C until their processing to accelerate freezing and halt DNA-destroying enzymatic processes.

### Sample processing and diagnostic methods

A small piece of gill tissue (2–5 mm^2^ of tissue) from each cockle was taken and stored at −20 °C for molecular assays. Genomic DNA from the cockle gill tissue was extracted using the chelex-100 extraction method^85,86^. The DNA of seabird faecal samples, as well as the sediment samples, were extracted by Qiagen QIAamp DNA Stool Kit, following the protocol of Zeale et al.^87^, with modifications from Dr James Nicholls (https://www.protocols.io/view/dna-extraction-from-avian-faeces-stored-in-ethanol-ve6e3he) and Sarah Davies (*pers comm*). Negative controls during DNA extraction were included to screen for potential contamination by prey DNA between samples and provide a higher degree of confidence in the assay protocol. Once extracted, the samples were used to determine the European cockle species (*Cerastoderma edule* (Linnaeus, 1758) or *Cerastoderma glaucum* (Poiret, 1789)) present and for subsequent pathological screening.

The quantity of target DNA remaining after storage, extraction and purification of the DNA, affects the successful amplification of that DNA from faecal material^52^. It should be highlighted that faecal DNA is often degraded, the risk of contamination is high, and PCR inhibitors may likely be co-extracted^52^. Therefore, the quantity and the quality of the extracted DNA from seabird faeces and sediment samples were established using NanoDrop 1000 Spectrophotometer to avoid the diagnosis of false negatives due to low DNA quantity and/or DNA of poor quality. DNA purity was assessed by determining the ratio of spectrophotometric absorbance of each extracted sample at 260/280 nm (A260/A280 ratio; an indicator of protein or phenol contamination). Pure DNA extraction should have an A260/A280 ratio ~ 1.8, higher values indicate the presence of RNA along with DNA.

Polymerase chain reaction (PCR) was carried out in the cockle, seabird faecal and sediment samples to amplify the nuclear DNA markers ITS-for / ITS Ce-R / ITS Cg-R to differentiate between the presence of *C. edule*, *C. glaucum* species or hybrids^88^ (Table 5). Amplification was conducted following the reaction mixture and thermocycling conditions, as well as the visualisation of the product, described in Albuixech-Martí et al.^35^.

**Table 5:** Description of PCR primer pairs showing sequences for each forward and reverse primer and expected product size.

Primer pair	Primer sequence (5’–3’)		Product size (bp)	Primer specificity
	Forward	Reverse		
ITS-for ITS Ce-R	GTT TCC GTA GGT GAA CCT G	AAG CAG CGA GAA GCC GTT C	190	*Cerastoderma edule*
ITS-for ITS Cg-R	GTT TCC GTA GGT GAA CCT G	AAT TCG CCA TCG TCG G	470	*Cerastoderma glaucum*
Vib1-F Vib2-R	GGC GTA AAG CGC ATG CAG GT	GAA ATT CTA CCC CCC TCT ACA G	120	*Vibrio* spp.
HAP-F1 HAP-R3	GTT CTT TCW TGA TTC TAT GMA	AKR HRT TCC TWG TTC AAG AYG A	350	Haplosporidia spp.
TAP-For TAP-Rev	ATC TAA CTA GCT GTC GCT AAC TCG T	CTT TCA AGA TTA CCC GGC TCT GC	165	*Minchinia tapetis*
MER-For MER-Rev	ATC TAA CTA GCT GTC ACT ATG GAA AA	ACG CAC ATT AAA GAT TGC CCA GCT CTT T	170	*Minchinia mercenariae*-like
OHVA OHVB	TGC TGG CTG ATG TGA TGG CTT TGG	GGA TAT GGA GCT GCG GCG CT	385	Ostreid herpesvirus type-1 and variants
MicIF1 MicIR1	GTG GAC GCT AGT CTC ACA GGT T	TTG CAC CAG AAG GTT TAC GAC ACA T	180	Microsporidia sp.1
MicIF2 MicIR2	ATG CAT GCG TAA GCG AAG CAG TTA T	TCT CTT GCA CCA GAA GGT TTA CGA C	180	Microsporidia sp.2

Standard PCR screening in cockle, seabird faecal and sediment samples was conducted using generic primers Vib1F/2R^89^ (Table 5) following the protocol modified by Vezzulli et al.^90^ (optimised by Davies, C. E.) for the detection of *Vibrio* genus. A total of 1 ml of DNA per individual was used in a total volume of 25 ml of the reaction mixture containing: 12.5 ml of Taq 2X Master Mix, 0.125 ml of for and reverse primers (100 mM) and 11.25 ml of ddH_2_O. Cycling conditions consisted of an initial denaturation of the sample at 94 °C for 10 min followed by 30 cycles of 94 °C for 30 s, 58 °C for 30 s, 72 °C for 60 s and a final elongation at 72°c for 10 min. Electrophoresis of the amplification products was conducted in a 2% agarose gel.

Standard PCR screening for haplosporidian detection was carried out in the cockle samples (n = 735), likewise in a subsample of seabird faecal and sediment samples (n = 168), using generic haplosporidian HAP-F1 and HAP-R3 primers that amplify small regions of the SSU rDNA of most haplosporidian parasites^91,92^ (Table 5). Amplification was conducted following the reaction mixture and thermocycling conditions, as well as the visualisation of the product, described in Albuixech-Martí et al.^35^.

Standard PCR screening for *Minchinia* spp. detection was carried out in the cockle samples that were positive for Haplosporidia by conventional PCR (n = 277), likewise in a subsample of seabird faecal and sediment samples (n = 168), using specific primers for *Minchinia tapetis* (TAP-For/Rev) and *Minchinia mercenariae*-like (MER-For/Rev)^35^ (Table 5) in the appropriate pairings and in separate PCR reactions. Amplification was conducted following the reaction mixture and thermocycling conditions, as well as the visualisation of the product, described in Albuixech-Martí et al.^35^.

Standard PCR screening for ostreid herpesvirus type-1 (OsHV-1) and variants was carried out in cockle samples (n = 735), likewise in a subsample of seabird faecal and sediment samples (n = 226), using specific primers OHVA/OHVB that amplify small products of the ORF4 gene in the OsHV-1 virus and its variants^93^ (Table 5). Amplification was conducted following the reaction mixture and thermocycling conditions described in Lynch et al.^93^. Electrophoresis of the amplification products was conducted in a 2% agarose gel.

Standard PCR using specific primers (MicIF1-MicIR1 and MicIF2-MicIR2, Table 5) (Lynch, unpublished data confirmed to be specific to Microsporidia spp. by Sanger sequencing) to detect two unidentified microsporidian species was carried out in cockle samples (n = 735), likewise in a subsample of seabird faecal and sediment samples (n = 116). Both reaction mixture and thermocycling conditions were the same as used with the PCR for OsHV-1, as well as the visualisation of the product.

Real-time quantitative PCR (qPCR) for detection and quantification of *Vibrio aestuarianus* in the cockle, seabird faecal and sediment samples that were positive for generic *Vibrio* screening was performed using specific primers for detection of the molecular chaperone *dnaJ* gene, following the protocol of McCleary and Henshilwood^63^ (Table 6)*.* All samples were performed in triplicates using 5 µl of DNA. C_t_ values were used to determine real-time PCR quantification and detection limits, a tested sample was considered positive if its mean C_t_ value was below 37.

**Table 6:** Description of qPCR primer pair and probe used in the study.

Primers	Sequence	Specificity
*dnaJ* f420 *dnaJ* r456 *dnaJ* p441 (probe)	GTGAAGGGACGGGTGCTAAG CCATGACAAGTGCCACAAGTCT FAM-AGGGCACGTCGGC-MGB	*Vibrio aestuarianus*

Direct Sanger sequencing was carried out on representative PCR products amplified from cockle samples, seabird faecal and sediment samples, using generic *Vibrio* primers to identify the species present in the samples. Genomic DNA from 10 selected cockle individuals and 11 faecal and sediment samples was isolated and purified using the QIAquick Gel Extraction Kit (QIAgen) prior to direct sequencing. Both the forward and reverse strands of DNA samples were sequenced commercially (Source Bioscience). Each sequence was matched against the National Center for Biotechnology Information (NCBI) nucleotide database with BLASTn (Basic Local Alignment Search Tool), which finds regions of local similarity between sequences to identify and confirm the DNA being detected in the PCRs.

### Statistical analyses

Statistical analysis was performed in R version 3.2.3. statistical software. *Vibrio* occurrence was modelled as pathogen presence/absence in each cockle, bird faecal sample and sediment sample. Pearson’s Chi-squared tests were used to determine whether the presence of *Vibrio* spp. was significantly different (p(Chisq) < 0.05) among the sample sites and throughout the year for each of the sample types (cockles, bird faecal samples and sediment). Fisher’s exact test was conducted when the frequencies of the pathogen occurrence in a site or season were < 4, to gain accuracy.

Collinearity between the different compartments—sediment, cockles, and shorebirds—was examined by pairs using non-parametric Kendall's Rank Correlation Tau (τ) with the *Vibrio* occurrence data in each compartment (p(τ) < 0.05). The correlation (p(τ) < 0.05) between the presence of the *C. edule* DNA and *Vibrio* DNA was also examined within two of the compartments, shorebirds and sediment.

## Supplementary Information

Supplementary Information.

## Data Availability

All data generated during this study are included in this article.
