# Supplementary material for: Connectivity dynamics in Irish mudflats between microorganisms including Vibrio spp., common cockles Cerastoderma edule, and shorebirds
Source: Sci Rep. 2021 Nov 12;11:22159. doi: 10.1038/s41598-021-01610-x (PMC8589998; doi:10.1038/s41598-021-01610-x)
Supplement: Supplementary file 1 — Supplementary Information. [file 41598_2021_1610_MOESM1_ESM.pdf]

Connectivity dynamics in Irish mudflats between microorganisms including *Vibrio* spp., common cockles *Cerastoderma edule*, and shorebirds

Sara Albuixech-Martí [1,*], Sharon A. Lynch [1,2], and Sarah C. Culloty [1,2,3]

[1] School of Biological, Earth and Environmental Sciences, University College Cork, Cork, VGV5+95, Ireland
[2] Aquaculture and Fisheries Development Centre, University College Cork, Cork, VGV5+95, Ireland
[3] MaREI Centre for Climate, Energy and Marine, Environmental Research Institute, University College Cork, Cork, VGV5+95, Ireland
[*]saraalbuixechmarti@ucc.ie

Supplementary Information

Supplementary Table S1: Seabird species, number, location and behaviour observed at each sample site (Ringaskiddy (Ringa.), Cuskinny (Cuski.), Youghal (Youg.), Dungarvan (Dung.), Annagassan (Annag.), and Cooley) by season.

Sites	Sampling dates	Tidal zonation	Bird species	Number of individuals	Behaviour
**Ringa.**	**Spring 2018**	Intertidal	Common gulls (*Larus canus*)	5	Loafing and swimming.
			Hooded crows (*Corvus cornix*)	1	Passively foraging.
	**Autumn 2018**	Intertidal	Common gulls (*Larus canus*)	2	Standing and swimming
			Black-headed gull (*Chroicocephalus ridibundus*)	1	
			Oystercatchers (*Haematopus ostralegus*)	3	Actively foraging and feeding
			Common redshank (*Tringa totanus*)	1	
			Curlew (*Numenius arquata*)	1	Flying
	**Winter 2018/19**	Intertidal	Common gulls (*Larus canus*)	3	Loafing and swimming.
			Oystercatchers (*Haematopus ostralegus*)	5	Actively foraging and feeding
			Curlew (*Numenius arquata*)	1	Passively foraging and preening.
			Brent gooses (*Branta bernicla*)	4	Swimming
	**Spring 2019**	Intertidal	Common gulls (*Larus canus*)	2	Loafing
			Great black-backed gulls (*Larus marinus*)	2	

**Cuski.**	**Spring 2018**	Intertidal	Common gulls (*Larus canus*)	4	Loafing
			Hooded crows (*Corvus cornix*)	Flock (~10)	Passively foraging and/or standing.
	**Autumn 2018**	Intertidal	Black-headed gulls (*Chroicocephalus ridibundus*)	Flock (40-50)	Preening, loafing, and passively foraging and feeding.
			Great black-backed gulls (*Larus marinus*)	2	
			Hooded crows (*Corvus cornix*)	1	Actively foraging and feeding
			Oystercatcher (*Haematopus ostralegus*)	1	
	**Winter 2018/19**	Intertidal	Common gulls (*Larus canus*)	Flock (20-30)	Passively foraging and/or standing.
			Hooded crows (*Corvus cornix*)	Flock (10-20)	
			Black-headed gulls (*Chroicocephalus ridibundus*)	4	
			Oystercatcher (*Haematopus ostralegus*)	1	Actively foraging and feeding
	**Spring 2019**	Intertidal	Black-headed gulls (*Chroicocephalus ridibundus*)	Flock (30-50)	Passively foraging and/or standing.
			Common gulls (*Larus canus*)	5	
			Great black-backed gulls (*Larus marinus*)	4	
			Hooded crows (*Corvus cornix*)	8	Actively foraging and feeding.
**Youg.**	**Summer 2018**	Intertidal	Red knot (*Calidris canutus*)	Flocks (10-20)	Actively foraging and feeding.
			Black-headed gulls (*Chroicocephalus ridibundus*)	2	Loafing
			Oystercatchers (*Haematopus ostralegus*)	2	Actively foraging and feeding.
			Curlews (*Numenius arquata*)	4	
			Grey heron (*Ardea cinerea*)	1	
	**Autumn 2018**	Intertidal and subtidal	Oystercatchers (*Haematopus ostralegus*)	3	Actively foraging and feeding.
			Common gulls (*Larus canus*)	4	Standing
			Curlew (*Numenius arquata*)	1	Actively foraging and feeding
			Bar-tailed godwits (*Limosa lapponica*)	2	
	**Winter 2018/19**	Intertidal and subtidal	Curlews (*Numenius arquata*)	2	Actively foraging and feeding.
			Oystercatchers (*Haematopus ostralegus*)	4	
			Bar-tailed godwit (*Limosa lapponica*)	1	
			Common gulls (*Larus canus*)	2	Loafing.
	**Spring 2019**	Intertidal	Oystercatchers (*Haematopus ostralegus*)	3	Actively foraging and feeding.
			Common gulls (*Larus canus*)	2	Standing.
			Grey heron (*Ardea cinerea*)	1	Actively foraging

**Dung.**	**Summer 2018**	Intertidal	Common gull (*Larus canus*)	1	Passively foraging and feeding.
			Oystercatchers (*Haematopus ostralegus*)	4	
			Hooded crow (*Corvus cornix*)	1	Standing
	**Autumn 2018**	Intertidal	Common gulls (*Larus canus*)	6	Passively foraging and/or standing.
			Great black-backed gulls (*Larus marinus*)	6	
			Black-headed gulls (*Chroicocephalus ridibundus*)	Sparse individuals (8) A flock (20-30)	
			Hooded crow (*Corvus cornix*)	1	
			Oystercatchers (*Haematopus ostralegus*)	4	Actively foraging and feeding.
			Common redshanks (*Tringa totanus*)	9	
	**Winter 2018/19**	Intertidal	Common gulls (*Larus canus*)	Flock (20-30)	Loafing
			Great black-backed gulls (*Larus marinus*)	Flock (20-30)	
	**Spring 2019**	Intertidal	Common gulls (*Larus canus*)	7	Loafing and preening.
			Great black-backed gulls (*Larus marinus*)	1	
			Oystercatchers (*Haematopus ostralegus*)	Flocks (~10)	Actively foraging and feeding.
			Brent gooses (*Branta bernicla*)	10	Swimming, loafing, preening.
**Annag.**	**Summer 2018**	Intertidal	Oystercatchers (*Haematopus ostralegus*)	9	Actively foraging and feeding.
			Curlews (*Numenius arquata*)	3	
			Black-headed gulls (*Chroicocephalus ridibundus*)	7	
			Dunlins (*Calidris alpina*)	Flock (10-20)	
			Common gulls (*Larus canus*)	3	Passively foraging and/or standing.
			Hooded crows (*Corvus cornix*)	1	
	**Autumn 2018**	Intertidal	Oystercatchers (*Haematopus ostralegus*)	Flocks (~10)	Actively foraging and feeding.
			Common redshank (*Tringa totanus*)	Flocks (~10)	
			Curlew (*Numenius arquata*)	1	
			Little egret (*Egretta garzetta*)	1	
			Common gulls (*Larus canus*)	1	Loafing.
	**Winter 2018/19**	Intertidal	Oystercatchers (*Haematopus ostralegus*)	Flocks (10-20)	Actively foraging and feeding.
			Black-tailed godwits (*Limosa limosa*)	Flocks (10-20)	
			Common redshank (*Tringa totanus*)	1	
			Curlew (*Numenius arquata*)	1	
	**Spring 2019**	Supratidal	Raven (*Corvus corax*)	1	Actively foraging

**Cooley**	**Summer 2018**	Intertidal	Common gulls (*Larus canus*)	4	Standing and loafing
			Black-headed gulls (*Chroicocephalus ridibundus*)	2	
			Great black-backed gull (*Larus marinus*)	1	
			Hooded crows (*Corvus cornix*)	2	
			Black-tailed godwits (*Limosa limosa*)	3	Actively foraging and feeding.
			Oystercatchers (*Haematopus ostralegus*)	Flocks (~10)	
	**Autumn 2018**	Intertidal	Common gulls (*Larus canus*)	3	Loafing and standing.
			Black-headed gulls (*Chroicocephalus ridibundus*)	2	
			Black-tailed godwits (*Limosa limosa*)	Flock (20-30)	Actively foraging and feeding.
			Grey heron (*Ardea cinerea*)	1	Standing
			Little egret (*Egretta garzetta*)	1	Passively foraging.
	**Winter 2018/19**	Intertidal	Oystercatchers (*Haematopus ostralegus*)	Flocks (~10)	Actively foraging and feeding.
			Black-tailed godwits (*Limosa limosa*)	5	
	**Spring 2019**	Intertidal and subtidal	Black-tailed godwits (*Limosa limosa*)	Flock (20-30)	Actively foraging and feeding.
			Common gulls (*Larus canus*)	Mixed flock (>50)	Actively foraging and feeding. Some loafing and preening.
			Mediterranean gulls (*Larus melanocephalus*)		
			Sandwich terns (*Sterna sandvicensis*)		

Supplementary Table S2: List of bird species observed in the study, their diet and status (based on www.birdwatchireland.ie).

Bird species	Diet	Status
Oystercatcher (*Haematopus ostralegus*)	The main food resource includes the larger invertebrates, particularly mussels and **cockles**.	Resident and winter visitor from Iceland and the Faeroes.
Curlew (*Numenius arquata*)	Feed mostly on invertebrates, particularly ragworms, crabs and molluscs including **cockles**[1].	Winter visitor to wetlands, as well breeding in small numbers in floodplains and boglands.
Bar-tailed godwit (*Limosa lapponica*)	Polychaete worms, particularly lugworms, form a large proportion of their diet. On the muddier estuaries, where lugworms may be absent, they take ragworms and **bivalves**.	Winter visitor to coastal estuaries from Russia and Scandinavia
Black-tailed godwit (*Limosa limosa*)	Feed on a range of invertebrates, including **bivalves**, polychaete worms and shore crabs.	Winter visitor from Iceland.
Red knot (*Calidris canutus*)	Feed predominantly on small bivalves, especially mussels, clams and **cockles**, and also on crustaceans.	Winter visitor from northern Greenland and the Queen Elizabeth Islands of high Artic Canada west to Prince Patrick Island. In spring and light summer some breeding plumage birds can be spotted.
Dunlin (*Calidris alpina*)	Feed predominantly on small invertebrates of estuarine mudflats, particularly polychaete worms and small molluscs, including **cockles**[2].	Summer visitor from NW Africa/SW Europe, winter visitor from Scandinavia to Siberia, passage migrant from Greenland (heading south to winter in Africa).
Common redshank (*Tringa totanus*)	Feed mainly on insects, earthworms, crustaceans and molluscs, including **cockles**[1].	Resident, winter visitor from Iceland and passage migrant (from Scandinavia/the Baltic breeding areas to African wintering areas).
Common gull (*Larus canus*)	Feed on a wide range of terrestrial and aquatic insects and invertebrates, also fish, carrion and rubbish. In sandy and muddy shores molluscs, including **cockles**, can serve as food[3].	Resident and winter visitor from Europe.
Black-headed gull (*Chroicocephalus ridibundus*)	Feed on mostly animal material, including a wide variety of insects, also earthworms, marine worms, molluscs, crustaceans, small fish and carrion. In sandy and muddy shores molluscs, including **cockles**, can serve as food[3].	Resident along all Irish coasts, with significant numbers arriving from the Continent in winter.
Great black-backed gull (*Larus marinus*)	Diet includes carrion, fish, molluscs, crustaceans, marine worms, insects, rodents, berries, and the adults, young, and eggs of other birds. On mudflats, they follow the retreating tide to capture worms and small **bivalves**.	Resident along all Irish coasts.
Mediterranean gull (*Larus melanocephalus*)	Feed on terrestrial and aquatic insects, marine molluscs, including **bivalves,** and fish, also offal and carrion.	Breeds in small numbers in the southeast. Winter visitor from northwest France, Belgium and the Netherlands.
Sandwich tern (*Sterna sandvicensis*)	Feed mainly on surface-dwelling fish, taken from shallow dive.	Summer visitor to all Irish coasts. Winters in small numbers in Galway Bay and Strangford Lough.
Hooded crow (*Corvus cornix*)	Diet includes seeds, insects, carrion, young birds and eggs. In coastal areas, will take crabs, **bivalve** and gastropod molluscs.	Common resident throughout Ireland.

Raven (*Corvus corax*)	May feed on practically anything, but mostly on insects and their larvae, worms and other subterranean invertebrates, also berries, grain, small mammals and birds.	Widespread resident throughout Ireland, especially in upland areas.
Brent goose (*Branta bernicla*)	During the winter, it feeds mostly on eel-grass.	Winter migrant from high-Arctic Canada. This population winters almost entirely in Ireland, with small numbers in parts of Britain and France.
Little egret (*Egretta garzetta*)	Takes a wide variety of animals including small fish, frogs, snails and insects	Resident along coasts and rivers throughout Ireland, but still scarce in the Midlands and the north-west.
Grey heron (*Ardea cinerea*)	Diet includes fish, amphibians, small mammals, insects and reptiles.	Common resident at wetlands, estuaries and along rivers throughout Ireland.

1. Goss-Custard, J. D. & Jones, R. E. Diets of redshank and curlew. *Bird Study* 23, 233-243 (1976).
2. Dit Durell, S. & Kelly, C. P. Diets of dunlin *Calidris-alpina* and gray plover *Pluvialis-squatarola* on the wash as determined by dropping analysis. *Bird Study* 37, 44-47 (1990).
3. Vernon, J. D. R. Feeding habitats and food of black-headed and common gulls. *Bird Study* 19, 173-186 (1972).
